# Studying Early Life Live-Attenuated influenza virus immune Responses (STELLAR): study protocol for an exploratory observational study of the nasal mucosal and systemic immune response in healthy children given an intranasal live-attenuated influenza vaccine

**DOI:** 10.1136/bmjopen-2025-114107

**Published:** 2026-06-25

**Authors:** Karen Kelleher, Reuben Bennett, Rebecca Jackson, Lauri Ivaska, Nisha Singh, Reyna Sara Quintero Barceinas, Emma Plested, Emma Francis, Yama F Mujadidi, Chidimma Nwankwo, Jack Francis-Hill, Amber Thompson, Carla Solórzano, Hannah Baughan, Xinxue Liu, Arnaud M Didierlaurent, Britta C Urban, Daniela M Ferreira, Dominic Kelly

**Affiliations:** 1 Oxford Vaccine Group, Department of Paediatrics, University of Oxford, Oxford, UK; 2 Center of Vaccinology, Department of Pathology and Immunology, University of Geneva, Geneva, GE, Switzerland

**Keywords:** Vaccination, IMMUNOLOGY, Paediatric infectious disease & immunisation

## Abstract

**Introduction:**

The mechanisms generating immunity involved in nasal vaccination for children are likely to differ significantly from both adult immunity and animal models. A deeper understanding is necessary for the development of future paediatric mucosal vaccines. Live-attenuated influenza vaccines (LAIVs) are licensed mucosal vaccines which confer protection in children against severe influenza. Beyond individual protection, mucosal vaccines can also limit transmission and colonisation, offering broader public health benefits than parenteral vaccination. We describe a protocol for an exploratory, observational study assessing systemic and mucosal responses to LAIV in healthy children.

**Methods and analysis:**

40 healthy participants aged 2–5 years inclusively will be recruited from the community. Researchers will conduct study visits in the participants’ homes and administer a single dose of their routine, yearly LAIV and collect blood, nasal cells, nasal fluid and saliva at baseline and at day 28 post-LAIV. They will have additional nasal fluid and saliva samples conducted by caregivers on days 1, 2, 3, 4, 6, 9, 14 and 21. They will complete a daily symptom report to detect symptoms due to vaccination as well as additional upper respiratory illnesses. Samples will be analysed to detect viral load for influenza virus strains included in LAIV, coinfection and colonisation by other pathogens, as well as assess the mucosal and systemic immune response to vaccination. This study aims to advance understanding of mucosal immunity mechanisms in children following live-attenuated influenza vaccination. This knowledge could help inform the development of future mucosal vaccines for children, potentially increasing both effectiveness, ease of delivery and tolerability in this vulnerable population.

**Ethics and dissemination:**

This protocol has been reviewed by the sponsor, collaborators and external peer reviewers. Ethical approval for this study has been obtained from the South Central - Hampshire B Research Ethics Committee (REC ref 24/SC/0251). Informed parental consent will be obtained prior to any study intervention. The results will be disseminated via publication in a peer-reviewed journal and presentation at international conferences.

**Study registration:**

ISRCTN87260269 (https://doi.org/10.1186/ISRCTN87260269).

**STRENGTHS AND LIMITATIONS OF THIS STUDY**

- This study uses serial, non-invasive mucosal sampling to track immune response kinetics following live-attenuated influenza vaccine (LAIV) vaccination in children, providing insights essential for evaluating vaccine efficacy and designing future mucosal vaccines for this demographic.
- By focusing on children aged 2–5, this study aims to uncover unique immunological insights into a high-risk demographic that cannot be replicated in adult or animal models, directly informing better protection strategies for this age group.
- This study will include testing for additional respiratory pathogens alongside LAIV vaccination, providing valuable context for interpreting vaccine-induced mucosal immune responses.
- While home sample collection by parents/caregivers enhances the study’s feasibility, limited prior experience may lead to variability in technique and sample quality between participants, potentially introducing heterogeneity in the dataset.

## Introduction

 Bacterial and viral lower respiratory infections are the fourth leading cause of morbidity and mortality worldwide, causing 2.4 million deaths per year.^1^ Children under 5 years old and adults over 65 years old are particularly at risk of severe or fatal respiratory diseases. Despite recent improvements, primarily due to increased vaccination coverage, lower respiratory tract infections remain the leading cause of death in children under 5 years old, after birth-related diseases.^2^ The human influenza viruses are associated with approximately 650 000 deaths per year and represent approximately 2% of all respiratory deaths,^3^ with coinfection with *Streptococcus pneumoniae, Staphylococcus aureus* and *Streptococcus pyogenes* all being associated with a higher mortality risk.^4^ Children under the age of 5 years account for approximately 100 000 of these deaths.^5^

Children are key spreaders of influenza and thus targeting vaccination towards children reduces community cases and fatalities more than targeting at-risk groups.^6^ This is likely because infected children have higher viral loads and shed virus in higher amounts and for longer periods than adults.^5^ Indeed, studies show that live-attenuated influenza vaccine (LAIV) vaccination of preschool-aged children results in reduced rates of influenza-like illness in older adults.^5 7^ Efficacy data regarding the use of LAIV consistently show that, in children, intranasally delivered LAIV is superior to intramuscular or subcutaneously delivered inactivated influenza vaccines with comparable safety data in children over 2 years old.^8^

Nasal vaccines against seasonal influenza are composed of three or four different live-attenuated influenza viruses. Europe and North America typically use the same strains and must be reformulated on an annual basis based on predictions from the Global Influenza Surveillance and Response System. LAIV is a cold-adapted, temperature-sensitive vaccine, and attenuated so that the viruses replicate in the nasopharynx with peak replication occurring at about 25°C. The viruses become inactivated at higher temperatures and are unable to replicate at core body temperatures, ensuring they cannot disseminate in the lower respiratory tract. While LAIV is highly effective at preventing disease in children and reduces transmission of influenza viruses in the community,^6^ it may not be as effective in adults.^8^ The latter is likely to be due to pre-existing immunity against influenza within the nasal mucosa neutralising the virus before an immune response can be generated.

Influenza viruses infect nasal epithelium, which triggers the induction of innate immunity and the release of cytokines, which in turn results in recruitment and activation of local immunity. Immune responses in mucosal tissue and systemic circulation are compartmentalised with some overlap between the two sites.^1^ Local immune responses are mainly generated in mucosal-associated lymphoid tissue, resulting in tissue-resident T and B cells. In children, vaccination with LAIV results in nasal cytokine production and increased IgA antibody titres in the nasal mucosa but only a limited increase in systemic levels of neutralising antibodies against influenza viruses.^8^ A recent study of LAIV in young adults showed that the induction of mucosal and systemic antibody responses is distinct from each other, with participants who developed Interleukin-33-mediated suppression of viral replication being less likely to develop a robust systemic antibody response although many still developed a mucosal IgA response. Vaccination with LAIV also resulted in activation of systemic CD8 and follicular helper cell responses in systemic circulation. Together, these data demonstrated both the compartmentalisation of, and interaction between, the mucosal and systemic immune system in response to LAIV.^7^

Unlike adults, young children have either absent or limited prior exposure to influenza viruses, and it is generally assumed that the absence of pre-existing immunity to influenza renders the LAIV more effective in children than in adults.^5^ However, the cellular response to LAIV, or indeed infection with wild-type influenza virus, in the nasal mucosa in children has not yet been studied but is critical for understanding the early immune responses resulting in control of nasal replication or susceptibility to wild-type virus dissemination into the lower respiratory tract.

### Study rationale

Understanding the kinetics of immune responses to LAIV and wild-type virus in the nasal mucosa, as well as the interplay between mucosal and systemic immunity, is key for identifying reliable correlates of protection. Such insights are critical for guiding the development of next-generation paediatric mucosal vaccines. This study targets children aged 2–5 years, a population chosen because children under five represent one of the highest-risk groups for severe illness and mortality from respiratory pathogens.^2 3 9^ Moreover, children in this age group typically have limited prior exposure to the influenza virus or LAIV, making them a uniquely important population in which to characterise vaccine-induced immune responses.

### Aims and objectives

This exploratory study aims to characterise the immune responses in the nasal mucosa and blood of children receiving the nasally administered LAIV (an infection-like stimulus). This manuscript is written in accordance with protocol version 2.0, dated 29 November 2024. Primary, secondary and exploratory objectives and outcome measures and time points are summarised in table 1.

**Table 1:** Objectives and outcome measures (see online supplemental material 1 for full details)

Objectives	Outcome measures
Primary objective	
Longitudinal assessment of LAIV strain-specific viral loads following vaccination with LAIV	Viral load of LAIV in nasal and saliva samples determined by qPCR
Secondary objectives	
Determine changes in antibody levels to influenza antigens in the mucosa	Antibody levels against influenza strains contained in LAIV determined by ELISA or comparable technical approach
To detect presence of nasal colonisation with pneumococcus or other respiratory pathogens	Detection of pneumococcus, most common respiratory viruses and other respiratory bacteria determined by microfluidic qPCR
To determine changes to influenza-specific antibodies in the systemic circulation	Antibody levels against the influenza strains contained in LAIV by ELISA or comparable technical approaches
To characterise transcriptional changes in mucosal immune cells in response to LAIV vaccination	Transcriptome analysis to identify gene signatures associated with response to vaccination in the nasal mucosa
Exploratory objectives	
To evaluate humoral responses and antigen-specific B cells to LAIV in systemic circulation	Analysis of antigen-specific B cells in systemic circulation using flow cytometry
To evaluate inflammatory responses to LAIV at the mucosa	Analysis of a range of inflammatory markers using Luminex multiplex panel or comparable technical approaches
To evaluate nasal cell populations before and after LAIV	Analysis of changes in cell populations in nasal mucosa by flow cytometry and related assays
To determine B cell receptor and T cell receptor full-length sequences in systemic circulation and compare with immune repertoire and expansion and/or contraction of clonotypes identified at single-cell level in the nasal mucosa	Analysis of scRNAseq and/or bulk transcriptome data to determine B cell receptor and T cell receptor sequences in the nasal mucosa
Comparison of immune response to LAIV infection over the life course (children, younger adults, older adults) by comparing data collected in this study to data from ECLIPSE (IRAS ID: 343692)	Meta-analysis of data generated in this study with comparable data generated in a parallel study on mucosal immune responses to vaccination with LAIV (NoseSpnLAIV Adults/ The ECLIPSE Study)
Comparison of immune and transcriptional parameters of LAIV vaccination with data obtained from children naturally infected with Influenza virus (symptomatic) by comparison with participants from a related study within the Nosevac consortium	Meta-analysis of data generated in this study with comparable data generated by collaborators in a hospital study (University of Geneva)
Evaluate the acceptability of study procedures including home sampling with participants and their parents including withdrawn participants	Analysis of anonymous voluntary survey issued via email

LAIV, live-attenuated influenza vaccine; qPCR, quantitative polymerase chain reaction.

## Methods and analysis

### Study design and setting

**Figure 1:**
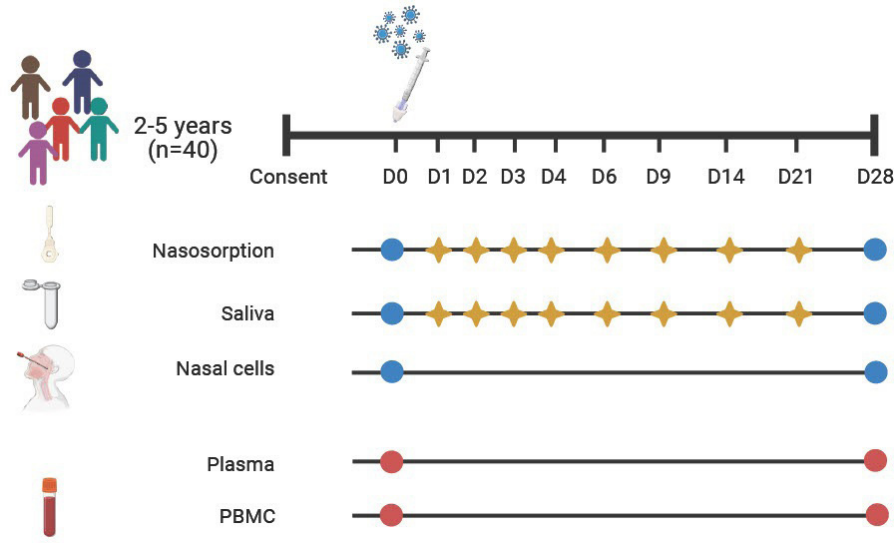
Sampling time points. Children are visited at home on day 0 and day 28 by the clinical team (circles). Parents and/or guardians will collect saliva and nasal lining fluid from children on days 1, 2, 3, 4, 6, 9, 14 and 21 (stars). PBMC: peripheral blood mononuclear cells

This is a single-site observational study that will enrol up to 40 children over two influenza seasons (September 2024 to February 2025 and September 2025 to February 2026) who are eligible for vaccination with LAIV as part of the National Vaccination Schedule. Children will be visited at home at enrolment and receive the LAIV vaccine as well as 28 days later for a follow-up visit by the clinical team, such that sample collection will be completed in February 2026. Recruitment of study participants will concentrate on Oxfordshire, England, and the surrounding areas to allow the timely transfer of biological samples to the laboratory. Parents will collect nasal lining fluid and saliva samples on days 1, 2, 3, 4, 6, 9, 14 and 21 (figure 1) following home sampling procedures demonstrated at visit 1 (V1) and in online supplemental material 2. Parents/guardians will also complete a daily symptom diary.

Finally, the acceptability of the study and home sampling procedure for children and their parents/guardians will be evaluated using an anonymous survey.

### Recruitment and eligibility

This study will recruit healthy children aged 2–5 years inclusively. Inclusion and exclusion criteria will be applied during the screening process at V1. A summarised list of these criteria is provided in Box 1.

Box 1 **Summarised inclusion and exclusion criteria (see online supplemental material 3 for full details)**

**Inclusion criteria**

- Child aged between 2 years and 5 years old at vaccination, eligible for yearly live-attenuated influenza vaccine (LAIV) vaccination in the UK, in good health.
- Parents/legal guardian(s) able to give informed consent, willing and able to comply with study procedures and over the age of 18.

**Exclusion criteria**

- Enrolled in another clinical trial or study (unless observational or at follow-up phase).
- Are taking daily medications that may affect the immune system.
- Have used inhaled, nasal, oral or intravenous steroid medications in the past 6 months.
- Have confirmed or suspected immunodeficiency/altered immunity.
- Have household contacts with severe immunodeficiency.
- Have already had an influenza vaccine in the current influenza season.
- Have a history of hypersensitivity/allergy to prior nasal spray influenza vaccine, or any constituent of the LAIV.
- Have a condition that needs salicylate therapy.
- Have unrepaired craniofacial malformations.
- Have a history of Guillain-Barré or Leigh syndromes.
- Unable to have products containing porcine gelatine or chicken eggs.
- Are a child of study site staff member.
- Or any other issue that, in the opinion of the study staff, may increase their/their family’s risk or adversely affect interpretation of study results.

**Temporary exclusion criteria**

- Have had or are having an exacerbation of asthma, or wheeze in the 72 hours before the LAIV is due.
- Have received any other vaccine in the past 14 days.
- Have any elective surgery, planned hospital admission or procedure requiring a general anaesthetic planned during the study period.
- Have a febrile illness in the previous 72 hours before the LAIV is due.

The study will be advertised through methods not limited to social media advertising, posters and an approved invitation letter. Potential participants’ parents can express interest in recruitment to the study by completing an online questionnaire or returning a reply slip by post. They will receive a participant information sheet containing detailed study information prior to the study team arranging a time to come to the participant’s home to complete consent, screening and study activities. Participants will be separately consented for their samples to be transferred to the Oxford Vaccine Centre (OVC) biobank. Participants will receive a £10 voucher at each study visit in recognition of their time and inconvenience. An example of the informed consent form can be found in online supplemental material 4.

### Study procedures

After consent is obtained and eligibility is assessed, study procedures are completed with the procedures and sampling schedule outlined in online supplemental material 5. Participants will be examined if directed by their medical history and have an axillary temperature performed to ensure it is not above 38 °C at the time of vaccination. Following this, sampling will be completed as described below, and then the LAIV will be administered.

### Sample collection

Nasal cells will be collected using flocked swabs. One sample per nostril will be obtained at D0 and D28. FloQSwabs flocked swabs are designed for nasal and mid-turbinate sampling. The brush-like texture of flocked swabs dislodges and collects cells for sampling and allows for more effective elution of the sample into the collection medium. To conduct the sampling, the swab will be inserted into the nostril with the participant’s head tilted slightly backwards, keeping the swab near the septum floor along the inferior turbinate and rotated several times while in contact with the inferior turbinate. The swab will be carefully withdrawn, and the sample stored in the collection tube as per the manufacturer’s instructions.

Blood sampling will be performed by trained, experienced staff. Up to 10 mL of blood will be collected at each visit (D0 and D28). The prior application of anaesthetic cream will reduce discomfort for the participant. A maximum of two phlebotomy attempts will be performed at each visit (D0 and D28). If phlebotomy is unsuccessful, the visit may be rescheduled for another day, a finger-prick sample may be attempted, or the procedure abandoned.

Nasosorption samples will be obtained using Synthetic Absorbent Matrix (SAM) strips, developed by Hunts Development Ltd (UK), which are similar to blotting paper. SAM strips absorb mucosal lining fluid while causing minimal discomfort to participants. Nasosorption strips will be held inside the nostril for up to 2 min, or until soaked through, or as tolerated by the participant. These will then be removed and placed in a microcentrifuge tube for storage.

Salivary sampling will be obtained using Oracol sample swabs (or similar), which were developed by Malvern Medical Developments (UK). Swabs will be removed from their packaging and held against the buccal mucosa for up to 2 min until soaked through. These will then be removed and placed back into their packaging with the sponge facing the microcentrifuge tube. The tube will be capped, and the stick of the sponge broken at the breakpoint, and the final cap replaced.

Home sampling will occur on days 1, 2, 3, 4, 6, 9, 14 and 21 post vaccination. Participants’ parents will be taught the technique for collecting SAM strips and salivary samples at visit 1 (V1) and instructed on labelling and storage of the samples in their own freezer until visit 2 (V2). Home samples will be collected at V2 onto dry ice before being processed at the laboratory. A temperature monitor and container for the storage of home samples will be provided by the study team. Temperature data will be evaluated for temperature deviations which may occur during storage in the home freezer following their collection at V2.

Symptom diaries will be issued to parents daily from the day of vaccination through to completion of V2. This will be issued as a Research Elctronic Data Capture (REDCap) survey sent via email, and it will collect information on the participant’s highest axillary temperature (on a thermometer given at V1), the presence and severity of any coryzal symptoms, altered feeding habits, diarrhoea, vomiting, irritability and drowsiness. The severity of each symptom will be graded by parents as per table 2. Any new medication or the need to seek medical attention will also be recorded. These data allow the detection of potential symptomatic intercurrent illness following the vaccination, which may change the interpretation of the data from collected samples. In case of issues completing the online diary, a paper backup diary is provided (online supplemental material 6).

**Table 2:** Symptom diary grading and description

None	No significant symptoms
Mild	Some symptoms, but no effect on usual activity
Moderate	Notable symptoms with some effect on normal activity
Severe	Symptoms that prevent normal activity

Upon completion of the V2, or following participant withdrawal, an optional REDCap end-of-study survey will be emailed to parents. Given that home SAM strip and salivary sampling are relatively new techniques, this survey will help to evaluate the acceptability of this, and the other study activities undertaken for parents and participants.

### Study objectives and measurements

The primary objective is to assess the influenza viral load of saliva and mucosal lining fluid across the sampling points throughout the study period. Secondary objectives include determining changes in antibody levels in the mucosa and systemic circulation, detecting changes in nasal colonisation with pneumococcus and other respiratory bacteria and viruses and characterising transcriptional changes following LAIV vaccination. A full list of the primary, secondary and exploratory objectives is included in Box 1. Samples will be stored for the duration of the study and thereafter, if consent is given, be transferred to the OVC biobank. All samples are processed by the OVC laboratory and stored on liquid nitrogen or in a −80 °C freezer per the laboratory analysis plan.

### Live-attenuated influenza vaccine vaccination

Following the collection of samples at V1, LAIV will be administered. In the UK, LAIV is routinely offered annually to children aged 2–17 years, as part of the national childhood immunisation programme. The LAIV being used in this study will be Fluenz, produced by AstraZeneca UK Limited. In the case of supply issues, an equivalent LAIV may be used. Fluenz comes as a suspension in a 0.2 mL pre-filled syringe for delivery by intranasal spray. There is an in-built divider to ensure that 0.1 mL will be delivered to each nostril. It contains three influenza virus strains: influenza A H1N1, influenza A H3N2 and influenza B Victoria. The exact strains included vary year to year and will comply with the WHO recommendation for the Northern Hemisphere and EU winter season. Each 0.2 mL dose contains 10^7+/−0.5^ fluorescent focus units. Administration of the vaccine will follow the manufacturer’s guidance, and the delivery protocol will be adapted from the Summary of Procuct Characteristics (SmPC) of the vaccine, which may change from season 1 to season 2. In most national programmes, 0.1 mL of LAIV is considered sufficient for protection (BCCDC, 2025). In this study, however, a participant receiving only 0.1 mL of vaccine will be excluded.

### Microbiological measurements

Viral load will be measured from nasal mucosal lining fluid (nasosorption sample) and salivary samples from V1, home samples and V2 through reverse transcription quantitative polymerase chain reaction (RT-qPCR). Detection of pneumococcus, other respiratory bacteria and common respiratory viruses will be assessed by microfluidic qPCR.

### Immunological measurements

Nasosorption samples and saliva will be used to monitor secretion of nasal inflammatory markers, antibody concentration and viral load for the influenza virus strains contained in the vaccine. They may also be tested for other common respiratory viruses/bacteria or other inflammatory signalling markers. Cytokine levels are measured by multiplex bead array.

Nasal cells will be analysed by flow cytometry to assess the cell-mediated immune response to vaccination, and RNA sequencing (RNA-seq) to assess the transcriptional changes associated with vaccination. In systemic circulation, immune responses to LAIV will be evaluated through analysis of plasma antibody levels. In addition, analysis of antigen-specific B cells or T cells by flow cytometry will be conducted depending on the results from the analysis of mucosal immune responses.

### Genetic measurements

To identify gene signatures associated with gene induction and regulation following LAIV vaccination, RNA-seq analysis is performed from nasal mucosa samples. Single-cell RNA-seq and/or bulk transcriptome data will be used to determine B cell and T cell receptor sequences in systemic circulation and the nasal mucosa in selected samples.

### Meta-analysis

These data will be compared by meta-analyses with two other studies within the Nosevac consortium. Differences in mucosal immune responses to vaccination across the lifespan will be assessed using samples from older participants in the parallel ECLIPSE study (IRAS ID: 343692). Comparisons with naturally acquired influenza infection will be conducted through meta-analysis of a cohort of age-matched hospitalised cases of paediatric influenza infection from collaborators at the University of Geneva.

### Statistical considerations

#### Sample size

As this is an exploratory study, the sample size was based on a feasibility assessment and ensuring that a sufficient number of participants will be recruited to analyse a range of nasal mucosal fluid and salivary time points. It is assumed that fewer samples will be collected than requested, and previous studies have indicated that approximately 75% of participants are likely to provide a sufficient number of samples.^10^ As such, we are vaccinating up to 40 children, expecting 30 participants will meet the minimum required number of samples for primary analysis (measuring viral load). A subset will also provide sufficient nasal cell samples for phenotyping and single-cell analysis as per other similar studies of respiratory pathogens.^11^ We will analyse all available data from study participants who were given the Fluenz vaccine.

#### Per-protocol population

For all objectives, analysis will only be carried out on those participants who receive the LAIV appropriately and who are part of the per-protocol population. For the primary outcome (LAIV viral load over time), the per-protocol population is defined as producing a nasal fluid sample at two or more time points prior to D9 post vaccination. For secondary outcomes, the per-protocol population is defined as having provided two blood samples, two home samples prior to D9 and at least the second nasal cell sample.

Exploratory end points will be analysed in the following populations: participants who have provided at least one nasal cell and blood sample and at least one post-vaccination nasorption sample. For some of the analyses, such as gene expression, a subset of participants will be selected for analysis based on clinical outcomes and availability of samples for the desired time points.

#### Statistical analysis

The analysis of the primary outcome involves the measurement of LAIV viral load over time. The LAIV load at different time points will be analysed in two ways:

1. The viral load will be summarised using the number, mean, geometric mean, SD, median, minimum and maximum at each time point. Log-transformed value will be analysed using a generalised estimating equation model, in which treatment, time and the interaction between treatment and time as independent factors after taking the correlation of participants into account. An exchangeable covariance structure will be used. The geometric mean ratio between groups together with their 95% CIs at each time point will be derived. If the interaction effect is non-significant, the interaction term will be dropped and the model refit, including only time and treatment fixed effects.
2. The area under the curve (AUC) of LAIV will be calculated by the trapezoidal rule from log10+1 transformed LAIV load results. The AUC will be summarised using the above descriptive statistics, and the log AUC will be analysed using a generalised linear model with a single factor of treatment. The geometric mean ratio between groups, together with their 95% CIs, will be derived.

For secondary and exploratory outcomes, the immune response data are expected to be highly skewed, and the data will be log-transformed prior to analysis. The geometric mean concentration and associated 95% CI will be summarised by computing the anti-log of the mean of the log-transformed data.

Continuous variables such as LAIV viral load and AUC over time will be summarised according to the number of subjects with non-missing data (n), mean, SD, median, minimum and maximum. Categorical variables such as symptoms and serious adverse events (SAEs) will be summarised according to the absolute frequency and percentage of subjects (%) in each category level. The denominator for the percentages is the number of subjects vaccinated with LAIV with data available, unless noted otherwise.

### Public and patient involvement

For this study, input was sought from the Oxford Vaccine Centre’s Patient and Public Involvement and Engagement (PPIE) group. A focus group involving three PPIE members was convened to discuss the study’s design and objectives. The group provided feedback on participant-facing materials, including information sheets, consent forms and recruitment documents, which was incorporated into the final versions. In addition, PPIE members contributed suggestions on recruitment approaches and advised on strategies for sharing study findings with the broader community.

## Ethics and dissemination

### Ethical and safety considerations

The study will be conducted in accordance with the current approved protocol, Good Clinical Practice guidelines, relevant regulations, the Declaration of Helsinki and local standard operating procedures. A risk assessment and monitoring plan has been prepared and will be reviewed throughout the study. Approved and relevant Standard Operating Procedures (SOPs) will be used at all clinical and laboratory sites. Ethical approval for this study has been obtained from the South Central - Hampshire B Research Ethics Committee and Health Research Authority on 12 August 2024; REC reference: 24/SC/0251. Parental informed consent will be sought by trained researchers prior to any study intervention. The university has a specialist insurance policy in place which will operate in the event of any participant suffering harm because of their involvement in research. The Strengthening the Reporting of Observational Studies in Epidemiology (STROBE) guideline^12^ was used in the development of this manuscript and the STROBE reporting checklist^13^ is included in online supplemental material 7.

### Risks

The general risks to participants from this study are very low. Participants will be counselled for risks from LAIV, including coryzal symptoms, anaphylaxis and wheeze, as well as pain and bleeding or bruising from venepuncture and nasal sampling. Potential participants at risk of more severe side effects will be excluded as per Box 1. Participants have access to a healthcare professional 24/7 in the event of illness throughout the study.

### Safety reporting

As this study is using LAIV in its licensed way, and all study procedures are considered low risk, it is not intended to report on adverse events (AEs) related to the LAIV. However, any suspected SAEs (defined as any AE that results in death, is life-threatening and requires hospitalisation or prolongation of existing hospitalisation, significant disability/incapacity or congenital anomaly/birth defects) will be assessed for relatedness and reported to the sponsor within 24 hours of notification. Reports of related and unexpected SAEs are submitted to the Health Research Authority (HRA) within 15 working days of the chief investigator becoming aware of the event.

### Other ethical considerations

To ensure no participant is inadvertently delayed in receiving their influenza vaccine, recruitment and administration of the vaccine will be timed so that participants who are found not to meet the enrolment criteria still have time to source their influenza vaccination through their primary care provider or school programme. We will, however, continue to enrol unvaccinated children as long as the vaccine is in date, unless this would lead to a delay in their vaccination.

### Data access and management

Consent will be documented on paper and recorded on the study database. All study data will be collected and recorded onto a secure REDCap database designed for the study—both investigator-collected data and parent/guardian-collected data (ie, the sample collection data, eDiary and eFeedback form). Parents/guardians have access to a backup paper diary in case of any inability to enter information online. The database has predefined data types and logic types for real-time data validation. Data collection and storage will be inspected throughout the study by the data management and monitoring teams. Participant data will be de-identified prior to reading or analysis. Any databases with participant-identifying details will be stored securely and only accessible by delegated study staff.

### Dissemination

Dissemination of the findings is planned for publication in peer-reviewed journals and at scientific international conferences. The investigators will be involved in reviewing drafts of the manuscripts, abstracts, press releases and any other publications arising from the study. The drafts may also be reviewed by the Nosevac consortium before dissemination.

All communication or publications concerning the project, including at a conference or seminar, shall acknowledge the parties involved, and the contribution by UK Research and Innovation under the Horizon Europe Guarantee Extension. Authorship will be determined in accordance with the International Committee of Medical Journal Editors (ICMJE) guidelines, and other contributors will be acknowledged.

## Supplementary material

10.1136/bmjopen-2025-114107 online supplemental file 1

10.1136/bmjopen-2025-114107 online supplemental file 2

10.1136/bmjopen-2025-114107 online supplemental file 3

10.1136/bmjopen-2025-114107 online supplemental file 4

10.1136/bmjopen-2025-114107 online supplemental file 5

10.1136/bmjopen-2025-114107 online supplemental file 6

10.1136/bmjopen-2025-114107 online supplemental file 7
