# Supplementary material for: Studying Early Life Live-Attenuated influenza virus immune Responses (STELLAR): study protocol for an exploratory observational study of the nasal mucosal and systemic immune response in healthy children given an intranasal live-attenuated influenza vaccine
Source: BMJ Open. 2026 Jun 25;16(6):e114107. doi: 10.1136/bmjopen-2025-114107 (PMC13311587; doi:10.1136/bmjopen-2025-114107)
Supplement: online supplemental file 1 [file bmjopen-16-6-s001.docx]

# Supplementary Material 1 - Primary, Secondary and Exploratory Objectives and Outcome Measures

In this study we propose to characterise the immune response in the nasal mucosa and in blood of children receiving LAIV (an infection-like stimulus).

|  | Objectives | Outcome Measures | Timepoint(s) |
| --- | --- | --- | --- |
| **Primary Objective** | Longitudinal assessment of viral load following vaccination in the nose | Viral load of LAIV from the nose and in saliva will be assessed by RT-qPCR | Nasosorption and saliva sampling on days 0, 1, 2, 3, 4, 6, 9, 14, 21 and 28 of the study. |
| **Secondary Objectives** | To determine changes in antibody levels to Influenza antigens in the mucosa over time | Measurement of antibody levels against the Influenza strains contained in LAIV by ELISA or comparable technical approaches | As per nasosorption sampling schedule above. |
|  | To detect presence of nasal colonisation with pneumococcus and other respiratory viruses and bacteria | Detection of pneumococcus, most common respiratory viruses and other respiratory bacteria will be assessed by microfluidic qPCR | As per nasosorption sampling schedule above. |
|  | To determine changes in antibody levels to Influenza antigens in systemic circulation over time | Measurement of antibody levels against the Influenza strains contained in LAIV by ELISA or comparable technical approaches | Blood at day 0 and day 28 post-vaccination |
|  | Characterise mucosal immune cells transcriptional changes (single cell and bulk RNA sequencing) in response to LAIV vaccination | RNAseq analysis to determine gene induction and regulation to identify gene signatures associated with response to vaccination in the nasal mucosa | Nasal samples in selected individuals |
| **Exploratory Objectives** | To evaluate humoral responses and antigen-specific B cells to LAIV in systemic circulation | Analysis of antigen-specific B cells in systemic circulation using flow cytometry | Blood at day 0 and day 28 post-vaccination |
|  | To evaluate inflammatory responses to LAIV at the mucosa | Analysis of a range of inflammatory markers using Luminex multiplex panel or comparable technical approaches | As per Nasosorption sampling schedule above. |
|  | To evaluate changes of nasal cell populations before and after LAIV vaccination | Analysis of changes in cell populations in nasal mucosa by flow cytometry and related assays | Nasal cell samples day 0 and day 28 |
|  | Characterise mucosal immune cells transcriptional changes (single cell and bulk RNA sequencing) in response to LAIV vaccination | RNAseq analysis to determine gene induction and regulation to identify gene signatures associated with response to vaccination in the nasal mucosa | Nasal samples in selected individuals |
|  | To determine B cell receptor and T cell receptor full length sequences at the single cell level to identify clonotypes enriched in the nasal mucosa. | Analysis of scRNAseq and/or bulk transcriptome data to determine B cell receptor and T cell receptor sequences in the nasal mucosa | Nasal samples in selected individuals |
|  | To determine B cell receptor and T cell receptor full length sequences in systemic circulation and compare with immune repertoire and expansion and/or contraction of clonotypes identified at single cell level in the nasal mucosa. | Analysis of scRNAseq and/or bulk transcriptome data to determine B cell receptor and T cell receptor sequences in systemic circulation and comparison of these data with those obtained from the nasal mucosa | Blood day 0 and day 28 in selected individuals |
|  | Comparison of immune response to LAIV infection over the life course (children, younger adults, older adults) | Meta-analysis of data generated in this study with comparable data generated in a parallel study on mucosal immune responses to vaccination with LAIV (NoseSpnLAIV Adults/ The ECLIPSE Study) | All data generated above |
|  | Comparison of immune and transcriptional parameters of LAIV vaccination with data obtained from children naturally infected with Influenza virus (symptomatic) by comparison with participants from a related study within the Nosevac consortium | Meta analysis of data generated in this study with comparable data generated by collaborators in a hospital study (University of Geneva). | All data generated above |
|  | Evaluate the acceptability of study procedures including home sampling with participants and their parents including withdrawn participants. | Analysis of anonymous voluntary survey issued via email through REDcap. | After the last D28 visit each season or after withdrawal from the study. |
