# Supplementary material for: Studying Early Life Live-Attenuated influenza virus immune Responses (STELLAR): study protocol for an exploratory observational study of the nasal mucosal and systemic immune response in healthy children given an intranasal live-attenuated influenza vaccine
Source: BMJ Open. 2026 Jun 25;16(6):e114107. doi: 10.1136/bmjopen-2025-114107 (PMC13311587; doi:10.1136/bmjopen-2025-114107)

**STELLAR - Home sampling instructions**

**Sample Collection Day Table:**

| Date | Day 1 | Day 2 | Day 3 | Day 4 | Day 6 | Day 9 | Day 14 | Day 21 |
| --- | --- | --- | --- | --- | --- | --- | --- | --- |
| **Sample type** |  |  |  |  |  |  |  |  |
| Nasosorption | X | X | X | X | X | X | X | X |
| Saliva | X | X | X | X | X | X | X | X |

**(Actual dates are mentioned in the ‘Appointment Card’ document)**

It is very important to take these samples at the same time each day. However, if you do forget to take your samples at the same time of day, please take your samples as soon as you remember (even if that is on a different day). Record the ACTUAL time and date the sample is taken – rather than the scheduled date – if different.

Below are some instructions to help you to take these samples. (This would have all been explained at your appointment). If you have any question, please feel free to contract the study team.

**Please remember to complete your e-diary to record the time and length of time the samples were taken**.

All used tubes and packaging can be disposed of in your household bin.

**Instructions for Sample collection:**

1. **Nasal (Nasosorption) sample instructions:**

**IMPORTANT:**

Wash your hands or use sanitizer gel, if you have it. If children are self-sampling, they also need to wash or sanitize their hands.

Make sure you do sample collection in a clean area (use a disinfectant wipe to clean the area e.g. kitchen table).

1. Set a timer on your phone for 2 minutes. The sample needs to be taken for up to 2 minutes or as long as tolerated by the child.
2. Tear open the foil packaging. Take the nasosorption strip out of the tube and hold it by its handle. Be careful not to contaminate or touch the strip (e.g. putting it on a table or touching your face (See picture (a)).
3. Place the strip in one nostril touching the inside of your child’s nose with the flat side against the nostril (See picture (b)) and press timer with the other hand. Keep the strip in place by pressing gently on the nostril for up to 2 minutes. Please time this accurately. If this is not possible (in the case of young children), record the time achieved in your diary. You may take samples from small children while sleeping to increase collection time.
4. Return strip to container (See picture (c)) and ensure lid is securely closed. (See picture (d)).
5. Fill in the diary, noting how long the sample was taken for.
6. Store in a storage container in your freezer at home (or in a fridge if you are not home, and place in freezer as soon as possible).
7. Please put the provided temperature logger in your freezer with your samples, this will be collected along with the samples at visit 2.


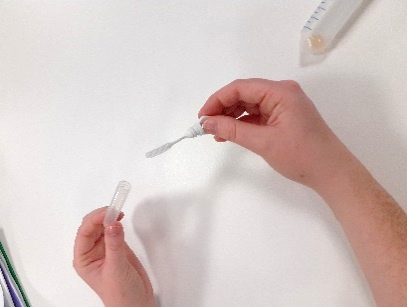


1. **Unscrew strip**


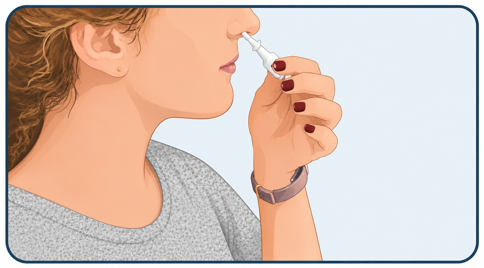


1. **Place strip in one nostril**

**
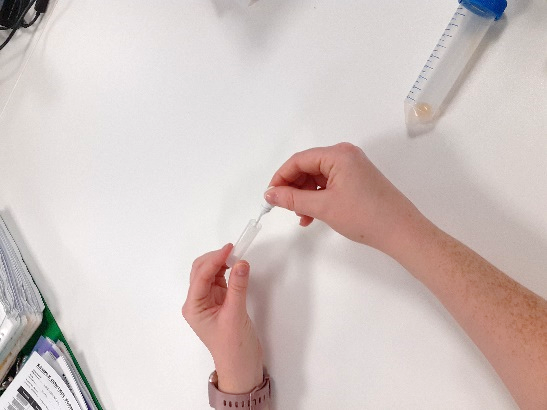
**

1. **Return strip to container**

**
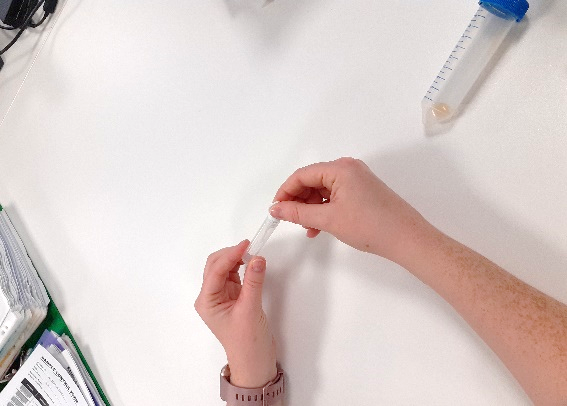
**

1. **Securely close container**
2. **Saliva sample instructions:**

**IMPORTANT:**

Wash your hands or use sanitizer gel, if you have it. If children are self-sampling, they also need to wash or sanitize their hands. Do this before helping infants, toddlers, and pre-schoolers to take their saliva sample (before touching the paediatric swab).

Make sure you do sample collection in a clean area (use a disinfectant wipe to clean the area e.g. kitchen table).

Please avoid drinking, eating, chewing gum, brushing teeth, or breastfeeding (in the case of infants) for 30 minutes before taking the saliva sample. We recommend doing this as soon as you get up in the morning.

1. Peel back protective package and remove the swab (See image 2 below). Do not use the swab if cuts or tears are present. If that is the case, use an extra swab provided and label with the child’s ID.
2. Carefully remove the swab device and take the handle end. Place the sponge end on the child’s tongue (or under the tongue if possible) and corners of the mouth (See image 3 below). Allow toddlers to chew or suck on the swab. Older children may wish to hold the swab themselves, just be careful they don’t remove from their mouth and touch anything with the swab.
3. Collect for up to 2 minutes** (as close to 2 minutes as possible) by resting the swab inside the mouth or collect in intervals by re-introducing the swab into the mouth as needed, until the lower third of the swab is saturated. Repeat as required.
4. Place the saturated end of the swab into the swab storage tube (See image 4 below). Snap off the handle at the breakpoint and re-attach screw cap.
5. Fill in the diary, noting how long the sample was taken for.
6. Store in a storage container in your freezer at home (or in a fridge if you are not home, and place in freezer as soon as possible).

****We are aware that 2 minutes may not be tolerated by young children, so we ask that you please keep the swab in your child’s mouth as close to 2 minutes as possible**

At the end of sampling, please wash your hands for 20 seconds with soap & warm water.


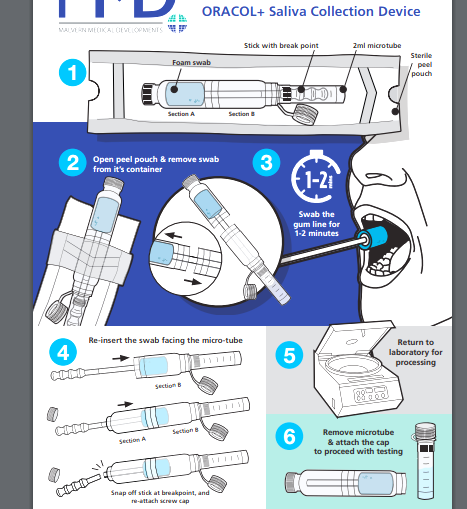

Supplement: online supplemental file 2 [file bmjopen-16-6-s002.docx]
