# Supplementary material for: Studying Early Life Live-Attenuated influenza virus immune Responses (STELLAR): study protocol for an exploratory observational study of the nasal mucosal and systemic immune response in healthy children given an intranasal live-attenuated influenza vaccine
Source: BMJ Open. 2026 Jun 25;16(6):e114107. doi: 10.1136/bmjopen-2025-114107 (PMC13311587; doi:10.1136/bmjopen-2025-114107)
Supplement: online supplemental file 3 [file bmjopen-16-6-s003.docx]

# Supplementary Material 4: Inclusion and Exclusion Criteria

## Inclusion Criteria

Children and Parents/guardian must satisfy all the following criteria to be eligible for the study:

- Children aged between 2 years and 5 years old (at vaccination) eligible for yearly LAIV vaccination in the UK, in good health.
- Parents/legal guardian(s) have capacity to give informed consent.
- Parents/legal guardian(s) are willing and able to comply with all study procedures.
- Parents/legal guardian(s) who are over 18 years of age and are able and willing to provide written informed consent for their child’s participation in the study.

## Exclusion Criteria

### Absolute Exclusion Criteria

Children may not participate in the study if any of the following apply:

- Are enrolled in another clinical trial/research study unless observational or follow-up phase (or at the discretion of the lead clinician)
- Are taking daily medications that may affect the immune system
- Are currently taking a steroid therapy (inhaled, nasal, oral or intravenous), or have been on these medications in the previous 6 months
- Are confirmed or suspected to have any disease or syndrome associated with altered immunity or immunodeficiency
- Have household contacts with a severe immunodeficiency (for example, someone who has had a recent bone marrow transplant)
- Have already had an influenza vaccine in the current influenza season
- Have a history of hypersensitivity to any of the following constituents – Sucrose, Dipotassium phosphate, Potassium dihydrogen phosphate, gelatin (porcine, Type A), arginine hydrochloride, monosodium glutamate monohydrate, gentamicin
- Have a history of an allergic reaction to a nasal spray flu vaccine in the past
- Are on or have a condition that needs salicylate therapy
- Have unrepaired craniofacial malformations
- Have a history of Guillain-Barré syndrome or Leigh syndrome
- The nasal spray vaccine contains small traces of gelatine derived from pigs (porcine gelatine). If the use of porcine gelatine in medical products is unacceptable, they will be excluded.
- The influenza strains for the nasal spray vaccine are cultured in chicken eggs. This does not increase the risk of anaphylaxis in children with egg allergy, but if the use of chicken eggs is unacceptable, they will be excluded.
- Are a child of a study site staff member
- Any other issue, in the opinion of the study staff, may:
- Put the children or their contact at risk because of participation in the study, or
- Adversely affect the interpretation of the study results

If they are excluded from the study for any of these reasons, they will be instructed to contact their GP or specialist medical practitioner to determine which influenza vaccine, if any, that they should receive.

### Temporary Exclusion Criteria

Children are temporarily excluded from participating if they:

- Are currently experiencing an exacerbation of asthma or wheeze symptoms when due the LAIV, including the increased use of relieving inhalers in the preceding 72 hours
- Have received any other vaccine within 14 days prior to the study vaccine
- Have scheduled elective surgery, planned admission or other procedures requiring general anaesthesia within the study period
- Have a febrile illness (axillary temperature ≥38.0°C) within the previous 72 hours of the scheduled vaccination
