# Supplementary material for: Studying Early Life Live-Attenuated influenza virus immune Responses (STELLAR): study protocol for an exploratory observational study of the nasal mucosal and systemic immune response in healthy children given an intranasal live-attenuated influenza vaccine
Source: BMJ Open. 2026 Jun 25;16(6):e114107. doi: 10.1136/bmjopen-2025-114107 (PMC13311587; doi:10.1136/bmjopen-2025-114107)
Supplement: online supplemental file 4 [file bmjopen-16-6-s004.docx]

| STELLAR- Studying Early-Life Live Attenuated influenza virus immune Response |
| --- |

Parent Informed Consent Form

**Participant identification number: ______________________________________________**

Child’s first name and surname: ______________________________ Child’s Initials: ___ ___

*Please* ***initial*** *in box*

*if you agree*

| 1. | I confirm that I have read the Study information booklet for the STELLAR study, version__.__ dated _ _ - _ _ _ - _ _ _ _. I have had the opportunity to consider the information, ask questions and have had these answered satisfactorily. |  | |
| --- | --- | --- | --- |
| 2. | I understand that my child’s participation is voluntary and that I am free to withdraw my child at any time without giving any reason, without their medical care or legal rights being affected. |  | |
| 3. | I understand that relevant sections of my child’s medical notes and data collected during the study may be looked at by individuals from the Oxford Vaccine group, University of Oxford and from regulatory authorities, where it is relevant to my child taking part in this research. I give permission for these individuals to have access to my child’s records. |  | |
| 4. | I agree that my child will donate blood, saliva, nasal cells and nasal fluid samples. I consider these samples a gift to the University of Oxford, and I understand that my child will not gain any direct personal or financial benefit from them. |  | |
| 5. | I agree to my child’s General Practitioner and Health Visitor team being informed of my child’s participation in the study. |  | |
| 6. | I agree to my child’s General Practitioner and/or other treating doctors and/or any other healthcare provider service being approached for additional information regarding medical and vaccination history and study staff to access my child’s NHS medical records, if required. |  | |
| 7. | I understand and agree that the Oxford Vaccine Group Data Management and IT Team will be able to view my email address. I understand it will be held securely on a server at the University of Oxford. |  | |
| 8. | I agree for my child to take part in this study. |  | |
| **Optional:** | | *Initial:* | |
| 9. | *I agree to be contacted about ethically approved research studies for which my child may be suitable. I understand that agreeing to be contacted does not oblige my child to participate in any further studies.* | Yes | No |
|  |  |  |  |
| 10. | *I agree to being contacted about studies in the future that are related to this study, and I understand that I would be under no obligation to take part in the future.* |  |  |
| 11. | *I agree to be contacted about the study survey. I understand that agreeing to be contacted does not oblige me to complete the survey.* |  |  |

| Name (PRINT NAME): __________________________________________________________________  Relationship to child (parent or legal guardian only): _________________________________________  Signature: _______________________________________________ Date: __ __ /__ __ __/__ __ __ __  (DD/MMM/YYYY) |
| --- |
| Study Nurse/Doctor’s name (PRINT NAME): _________________________________________________  Signature: _______________________________________________ Date: __ __ /__ __ __/__ __ __ __  (DD/MMM/YYYY) |

*Original to be retained at site in the case report form (CRF); second copy to be given to the parent/guardian.*
