# Supplementary material for: Studying Early Life Live-Attenuated influenza virus immune Responses (STELLAR): study protocol for an exploratory observational study of the nasal mucosal and systemic immune response in healthy children given an intranasal live-attenuated influenza vaccine
Source: BMJ Open. 2026 Jun 25;16(6):e114107. doi: 10.1136/bmjopen-2025-114107 (PMC13311587; doi:10.1136/bmjopen-2025-114107)
Supplement: online supplemental file 5 [file bmjopen-16-6-s005.docx]

**Supplementary Table 1**: **Study procedures and sampling schedule:**

| **Study Visit** | **D0***  **(V1)** | D1 | D2 | D3 | D4 | D6 | D9 | D14 | D21 | **D28 (V2)** | **End of Study** |
| --- | --- | --- | --- | --- | --- | --- | --- | --- | --- | --- | --- |
| **Day post vaccine** | 0 | 1^ | 2^ | 3^ | 4^ | 6^ | 9^ | 14^ | 21^ | 28-35 |  |
| **Enrolment** | | | | | | | | | | | |
| Consent (Written)* | X |  |  |  |  |  |  |  |  |  |  |
| Consent (Verbal) |  |  |  |  |  |  |  |  |  | X |  |
| Study information***** | X |  |  |  |  |  |  |  |  |  |  |
| **Assessments** | | | | | | | | | | | |
| Clinical Exam (If indicated) | X |  |  |  |  |  |  |  |  |  |  |
| Vital Signs | X |  |  |  |  |  |  |  |  | X |  |
| Nasosorption | X | X | X | X | X | X | X | X | X | X |  |
| Nasal cells | X |  |  |  |  |  |  |  |  | X |  |
| Saliva | X | X | X | X | X | X | X | X | X | X |  |
| Blood (up to 10 ml) | X |  |  |  |  |  |  |  |  | X |  |
| Symptom Diary | Daily until V2 |  |  |  |  |  |  |  |  |  |  |
| Survey (Optional)** |  |  |  |  |  |  |  |  |  |  | X |
| **Interventions** | | | | | | | | | | | |
| LAIV | X |  |  |  |  |  |  |  |  |  |  |
| *D0 may be split over 2 visits if parents/legal guardians cannot accommodate consent process and sampling on same date, or to provide enough time to parent/guardian to consider participation, or for recruitment purposes to have participants screened and consented ahead of receiving the LAIV vaccine. ** Survey will be sent after D28 or after withdrawal from the study. | | | | | | | | | | | |
