# Supplementary material for: Studying Early Life Live-Attenuated influenza virus immune Responses (STELLAR): study protocol for an exploratory observational study of the nasal mucosal and systemic immune response in healthy children given an intranasal live-attenuated influenza vaccine
Source: BMJ Open. 2026 Jun 25;16(6):e114107. doi: 10.1136/bmjopen-2025-114107 (PMC13311587; doi:10.1136/bmjopen-2025-114107)
Supplement: online supplemental file 6 [file bmjopen-16-6-s006.docx]

# STELLAR – Back-up paper diary

**Participant’s number: ______________________________ Participant’s initials: ___ ___**  **(D0)** **Vaccination Date: _ _ /_ _ _ /_ _ _ _**

This diary is to be completed after the **Influenza vaccine has been given**.

Between Day 0 and Day 28 (+7 days), we ask you to record:

- Episodes of illnesses
- Any new medications taken
- Any visits to the GP or healthcare provider
- Temperature (to 1 decimal place) – if taken due to parental suspicion of illness
- General symptoms
- Log of home sampling – **please see end of diary for the log**

**Please note:**

- Day 0 is the day of the vaccination

**If your child is admitted to hospital for any reason, or you are concerned about your child’s health, please contact the study team as soon as possible:**

**In office hours (9am-5pm) - XXXXXXXXXXXXX Out of** **hours XXXXXXXXXXX**

**Participant’s number: ___________________________**

**Temperature**

Please record on this chart if your child feels hot or their temperature is greater than 38°C at any time during the day. Write down the highest temperature reading of the day, and the time when it was taken.

| **Date:** | **Time:** | **Highest temperature recorded on this date:** |
| --- | --- | --- |
|  |  |  |
|  |  |  |
|  |  |  |
|  |  |  |
|  |  |  |
|  |  |  |
|  |  |  |
|  |  |  |
|  |  |  |

**Participant’s number: ___________________________**

**General Symptoms, page 1**

|  | Day 0 | Day 1 | Day 2 | Day 3 | Day 4 | Day 5 | Day 6 | Day 7 | Symptom end date (if after day 7) |
| --- | --- | --- | --- | --- | --- | --- | --- | --- | --- |
| ***Cough/Sneezing/Runny nose*** *–* please circle  **None** – No significant cough or cold symptoms  **Mild** – Some coughing, runny nose and/or sneezing but no effect on normal activity  **Moderate** – Persistent cough, runny or blocked nose with an effect on normal activity  **Severe** – Severe and persistent cough, runny or blocked nose with a large effect on normal activity | None  Mild  Moderate  Severe | None  Mild  Moderate  Severe | None  Mild  Moderate  Severe | None  Mild  Moderate  Severe | None  Mild  Moderate  Severe | None  Mild  Moderate  Severe | None  Mild  Moderate  Severe | None  Mild  Moderate  Severe | _ _/_ _ _/_ _  DD/MMM/YY |
| ***Change in Feeding/Eating Habits*** *–* please circle  **None** – No change to feeding and no effect on normal activity  **Mild** - Feeding/eating less than usual/ no effect on normal activity  **Moderate** – Feeding/eating less than usual (for 1 -2 meals) with an effect on normal activity  **Severe** - Not feeding/eating at all | None  Mild  Moderate  Severe | None  Mild  Moderate  Severe | None  Mild  Moderate  Severe | None  Mild  Moderate  Severe | None  Mild  Moderate  Severe | None  Mild  Moderate  Severe | None  Mild  Moderate  Severe | None  Mild  Moderate  Severe | _ _/_ _ _/_ _  DD/MMM/YY |

**Participant’s number: ___________________________**

**General Symptoms, page 2**

|  | Day 0 | Day 1 | Day 2 | Day 3 | Day 4 | Day 5 | Day 6 | Day 7 | Symptom end date  (if after day 7) |
| --- | --- | --- | --- | --- | --- | --- | --- | --- | --- |
| ***Diarrhoea*** – please circle  **None** – No change to stools  **Mild** – Looser stools than usual  **Moderate** – Frequent runny stools without much solid material  **Severe** – Multiple liquid stools without much solid material | None  Mild  Moderate  Severe | None  Mild  Moderate  Severe | None  Mild  Moderate  Severe | None  Mild  Moderate  Severe | None  Mild  Moderate  Severe | None  Mild  Moderate  Severe | None  Mild  Moderate  Severe | None  Mild  Moderate  Severe | _ _/_ _ _/_ _  DD/MMM/YY |
| ***Irritability/fussiness*** – please circle  **None** – Not crying more than usual / no effect on normal activity  **Mild** – Crying more than usual / no effect on normal activity  **Moderate** – Crying more than usual / interferes with normal activity  **Severe** – Crying that cannot be comforted / prevents normal activity | None  Mild  Moderate  Severe | None  Mild  Moderate  Severe | None  Mild  Moderate  Severe | None  Mild  Moderate  Severe | None  Mild  Moderate  Severe | None  Mild  Moderate  Severe | None  Mild  Moderate  Severe | None  Mild  Moderate  Severe | _ _/_ _ _/_ _  DD/MMM/YY |

**Participant’s number: ___________________________**

**General Symptoms, page 3**

|  | Day 0 | Day 1 | Day 2 | Day 3 | Day 4 | Day 5 | Day 6 | Day 7 | Symptom end date  (if after day 7) |
| --- | --- | --- | --- | --- | --- | --- | --- | --- | --- |
| ***Drowsiness*** – please circle **None** - No drowsiness (normal behaviour)  **Mild** - Drowsiness easily tolerated  **Moderate** - Drowsiness that interferes with normal activity  **Severe** - Drowsiness that prevents normal activity | None  Mild  Moderate  Severe | None  Mild  Moderate  Severe | None  Mild  Moderate  Severe | None  Mild  Moderate  Severe | None  Mild  Moderate  Severe | None  Mild  Moderate  Severe | None  Mild  Moderate  Severe | None  Mild  Moderate  Severe | _ _/_ _ _/_ _  DD/MMM/YY |
| ***Vomiting –*** please circle  **None** – No vomiting  **Mild** – 1-2 episodes without interfering with routine  **Moderate** – Several episodes and cannot keep any food down  **Severe** – Frequent episodes and not feeding/eating at all | None  Mild  Moderate  Severe | None  Mild  Moderate  Severe | None  Mild  Moderate  Severe | None  Mild  Moderate  Severe | None  Mild  Moderate  Severe | None  Mild  Moderate  Severe | None  Mild  Moderate  Severe | None  Mild  Moderate  Severe | _ _/_ _ _/_ _  DD/MMM/YY |

**Participant’s number: ___________________________**

**Episodes of illness**

Please record details in the table below if your child

- becomes unwell between vaccination and the next study visit
- is seen by a doctor, nurse or other medical caregiver, for any reason

| **Date** | **Problem** | **Medical care sought** |  | | **Were any new medicines started?** |
| --- | --- | --- | --- | --- | --- |
|  |  |  | **YES** | **NO** |  |
| Date child became unwell:  ____/____/____    Date child recovered:  ____/____/____ |  | Phoned for advice (GP/practice nurse/NHS 111)? |  |  | **YES / NO**  **If YES, please complete the table below** |
|  |  | Seen by a GP/practice nurse/other medical caregiver? |  |  |  |
|  |  | Seen in A+E? |  |  |  |
|  |  | Admitted to hospital?  If your child is admitted to hospital for this illness, please contact the study nurse or doctor as soon as convenient |  |  |  |
|  |  |  |  |  |  |

**Participant’s number: ___________________________**

If your child is started on any new medications, please complete the table below:

(Please note that paracetamol or ibuprofen medicines given in the first 21 days after vaccination should be recorded in the tables on the “temperature” pages above, rather than in the table on this page.)

| Medication | Reason for medication | Dose and how it is given (*e.g.*by mouth/inhaler/injection) | Start date | End date |
| --- | --- | --- | --- | --- |
|  |  |  |  |  |
|  |  |  |  |  |
|  |  |  |  |  |
|  |  |  |  |  |
|  |  |  |  |  |

| **Sample collection day** | **Date samples**  **collected** | 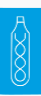  **Nasosorption sample taken**? | **Time taken** | 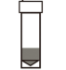  **Saliva sample taken**? | **Time taken** |
| --- | --- | --- | --- | --- | --- |
| **Day 1** | __ __ /__ __ __ /__ __ | 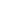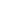Yes             No | __ __ : __ __    How long was sample  taken for ______(secs) | 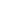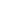Yes             No | __ __ : __ __    How long was sample  taken for (mins/secs) |
| **Day 2** | __ __ /__ __ __ /__ __ | 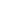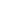Yes             No | __ __ : __ __    How long was sample  taken for ______(secs) | 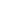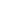Yes             No | __ __ : __ __    How long was sample  taken for ______(mins/secs) |
| **Day 3** | __ __ /__ __ __ /__ __ | 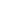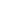Yes             No | __ __ : __ __    How long was sample  taken for ______(secs) | 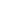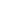Yes             No | __ __ : __ __    How long was sample  taken for(mins/secs) |
