# Supplementary material for: Studying Early Life Live-Attenuated influenza virus immune Responses (STELLAR): study protocol for an exploratory observational study of the nasal mucosal and systemic immune response in healthy children given an intranasal live-attenuated influenza vaccine
Source: BMJ Open. 2026 Jun 25;16(6):e114107. doi: 10.1136/bmjopen-2025-114107 (PMC13311587; doi:10.1136/bmjopen-2025-114107)
Supplement: Supplementary file 8 [file bmjopen-2025-114107.reviewer_comments.pdf]

# PEER REVIEW HISTORY

BMJ Open publishes all reviews undertaken for accepted manuscripts. Reviewers are asked to complete a checklist review form and are provided with free text boxes to elaborate on their assessment. These free text comments are reproduced below.

## ARTICLE DETAILS

### Title (Provisional)

STudying Early Life Live-Attenuated influenza virus immune Responses (STELLAR): study protocol for an exploratory observational study of the nasal mucosal and systemic immune response in healthy children given an intranasal live-attenuated influenza vaccine

### Authors

Kelleher, Karen Jean; Bennett, Reuben David Robert; Jackson, Rebecca Imogen; Ivaska, Lauri; Singh, Nisha; Quintero Barceinas, Reyna Sara; Plested, Emma; Francis, Emma; Mujadidi, Yama; Nwankwo, Chidimma; Francis-Hill, Jack; Thompson, Amber; Solórzano, Carla; Baughan, Hannah; Liu , Xinxue; Didierlaurent, Arnaud M.; Urban, Britta; Ferreira, D.M.; kelly, dominic f

---

## VERSION 1 - REVIEW

---

| | |
|---|---|
| **Reviewer** | **1** |
| **Name** | **Thomas, Roger** |
| **Affiliation** | **University of Calgary, Family Medicine** |
| **Date** | **03-Apr-2026** |
| **COI** | **None** |

---

A very important and well planned study. My comments are confined to the % of samples you will actually obtain and how you might accomplish a higher sampling rate.

“They will have blood, nasal cells, nasal-fluid and saliva collected at baseline and at day 28 post-LAIV. They will have additional nasal fluid and saliva samples conducted by caregivers on days 1, 2, 3, 4, 6, 9, 14 and 21. They will complete a daily symptom report to detect symptoms due to vaccination as well as additional upper-respiratory illnesses.”

[Do you have data from prior studies on the refusal rates over time by children and/or their parents to repeated especially nasal samplings? And daily symptom reports]

“Ethical approval for this study has been obtained from the South Central - Hampshire B Research Ethics Committee (REC ref 24/SC/0251).” [Why was approval not sought from the ethics committee relevant to Oxford University?]

“Unlike adults, young children have either absent or limited prior exposure to influenza viruses, and it is generally assumed that the absence of pre-existing immunity to influenza renders the LAIV more effective in children than adults [5]. However, the cellular response to LAIV, or indeed infection with wild-type influenza virus, in the nasal mucosa in children has not yet been studied but is critical for understanding the early immune responses resulting in control of nasal replication or susceptibility to wild-type virus dissemination into the lower respiratory tract.”

[This is your key message. Have you decided how you will motivate parents with this concept you wish to test?]

[Your primary and secondary objectives could be key motivators for parents, particularly if you could inform them promptly of the results for their child and with an interesting graphic for the child they could colour. Children find an activity motivating. Could this be done?]

Methods

“This is a single site observational study that will enrol up to 40 children over two influenza seasons.”

You are actually expecting 30 children. “It is assumed that fewer samples will be collected than requested, and previous studies have indicated that approximately 75% of participants are likely to provide a sufficient number of samples[10]. As such, we are vaccinating up to 40 children, expecting 30 participants will meet the minimum required number of samples for primary analysis (measuring viral load). A subset will also provide sufficient nasal cell samples for phenotyping and single-cell analysis as per other similar studies of respiratory pathogens.”

[Why did you decide on 40? I realise that a power computation for 40 children is not indicated for a protocol study, but should you do a larger appropriately powered study to guide the major subsequent RCT?]

---

## VERSION 1 - AUTHOR RESPONSE

**Reviewer Prof. Roger Thomas,**

Thank you for your positive evaluation of our study. We will address your questions below.

**Do you have data from prior studies on the refusal rates over time by children and/or their parents to repeated especially nasal samplings? And daily symptom reports**

We have considerable experience in taking blood samples from children of all ages for research. The previous work by Prof Ferreira in Liverpool “*Assessing the use of minimally invasive self-sampling at home for long-term monitoring of the microbiota within UK families”* by Nikolaou et al 2023 demonstrated reasonable tolerance of these procedures in children including under the age of five years. We extrapolated from this work to ensure that we will obtain at least 20 complete sample sets.

In other vaccine studies previously conducted at OVG, we usually ask parents to log into a website to record symptoms once per day, and they typically have access to a paper version, too. a paper version of the diary if they struggle to use the online version.

**Why was approval not sought from the ethics committee relevant to Oxford University?**

We usually submit applications for ethical approval to a regional (South-East) ethics committee based on availability to process the application.

**"Unlike adults, young children have either absent or limited prior exposure to influenza viruses, and it is generally assumed that the absence of pre-existing immunity to influenza renders the LAIV more effective in children than adults [5]. However, the cellular response to LAIV, or indeed infection with wild-type influenza virus, in the nasal mucosa in children has not yet been studied but is critical for understanding the early immune responses resulting in control of nasal replication or susceptibility to wild-type virus dissemination into the lower respiratory tract."**
**This is your key message. Have you decided how you will motivate parents with this concept you wish to test?**

We usually rely on parents/guardians to enrol their children into vaccine studies because they have an active interest in research and understand that there is no direct benefit to their child. However, they often want wish to contribute to research to improve vaccines for children in the future including development of nasal vaccines.

**Your primary and secondary objectives could be key motivators for parents, particularly if you could inform them promptly of the results for their child and with an interesting graphic for the child they could colour. Children find an activity motivating. Could this be done?**

The samples, to a large degree, will be extracted and frozen on the day of collection and stored for group analysis at a later date. At OVG, we also have a policy to provide study participants or their parents/guardians with published or overall results but not personalised results (unless clinically relevant). A generic colouring sheet could be considered of the overall pattern from this trial for future ones. In general, a play therapist accompanies the clinical team during study visit to provide entertainment and we give the children certificates for completion of the study.

**Why did you decide on 40? I realise that a power computation for 40 children is not indicated for a protocol study, but should you do a larger appropriately powered study to guide the major subsequent RCT?**

As this study is rather exploratory, the numbers were based upon estimates of feasibility to achieve usable results and form the basis for future studies. The type of analysis to be undertaken on nasal samples will provide high-dimensional multiset data for which complete sample set from 20 children are sufficient to achieve our aims. In addition, the results of this

study will be analysed alone but also compared to a very similiar study in children hospitalised with Influenza using the same methods.
